# Human limbal niche cells are a powerful regenerative source for the prevention of limbal stem cell deficiency in a rabbit model

**DOI:** 10.1038/s41598-018-24862-6

**Published:** 2018-04-26

**Authors:** Guigang Li, Yuan Zhang, Subo Cai, Ming Sun, Juan Wang, Shen Li, Xinyu Li, Sean Tighe, Shuangling Chen, Huatao Xie, Yingting Zhu

**Affiliations:** 1 Department of Ophthalmology, Tongji Hospital, Tongji Medical College, Huazhong University of Science and Technology, Wuhan, 430030 China; 2 Research and Development Department, Tissue Tech, Inc, Miami, FL 33126 USA; 3 Department of Ophthalmology, Union Hospital, Tongji Medical College, Huazhong University of Science and Technology, Wuhan, 430030 China

## Abstract

In this article, human limbal niche cells (LNC) or bone marrow derived mesenchymal stem cells (BMMSC) were used to prevent limbal stem cell deficiency (LSCD) in an alkali burn rabbit model and their results were compared. The epithelial cell defect area, corneal neovascularization, and the print cell cytometry were quantified to grade the severity of LSCD. Three months after the alkali burn, a partial LSCD was observed in the control group (no treatment) indicated by chronic corneal epithelial defects, positive corneal fluorescein staining, neovascularization and goblet cell migration. In contrast, the severity of LSCD in both the LNC and BMMSC transplantation groups was dramatically reduced as shown by smaller epithelial cell defects, decreased fluorescein sodium staining, decreased neovascularization and decreased goblet cell density. Interestingly, the LNC group was shown to more effectively prevent LSCD than the BMMSC group. Further analysis indicated subconjunctivally transplanted LNCs were more powerful than BMMSCs to prevent LSCD, at least partially, due to increased activation of SCF-c-Kit signal. We conclude that LNCs are a more powerful resource than BMMSCs to prevent LSCD in an alkali burn rabbit model, at least partially due to increased activation of SCF signaling.

## Introduction

Although corneal transplantation is a standard treatment for serious cornea diseases, many patients are not able to recover from blindness due to limbal stem cell deficiency (LSCD). The causative factors for LSCD include a variety of etiologies such as chemical or thermal burns, Stevens Johnson syndrome, Sjogren’s syndrome, multiple surgeries and other chronic ocular surface inflammatory processes. LSCD may lead to delayed cornea epithelialization, cornea conjunctivalization, and corneal opacification and as a result the vision becomes severely impaired^1^. Over the past decades, several medical treatments for LSCD have been reported including amniotic membrane transplantation, autograft LSC and oral mucosa transplantation, allograft LSC and oral mucosa transplantation, and bone marrow derived mesenchymal stem cells (BMMSC) or epithelial stem cells derived from corneal epithelial cells. However, there is still no optimal treatment probably due to lack of knowledge of the underlying mechanisms during LSCD occurrence and recovery^2,3^.

Nowadays it is increasingly popular to use stem cell (SC) treatment because they have the ability to self-renew and adopt fate decisions which may promote corneal surface reconstruction and healing. For example, the corneal epithelium may renew continuously due to a population of epithelial SCs located at the “limbal palisades of Vogt” between the cornea and the conjunctiva^4,5^. Furthermore, cumulative evidence has shown that self-renewal and fate decisions of SC are regulated by a “niche”, which is a specialized microenvironment around the SC^6,7^. The clinical importance of the limbal niche containing adult mesenchymal stem cells (MSC) has been recognized for decades as the treatment strategy is aimed at restoring and preserving the niche for successful patient outcome^1^.

MSCs are a group of multipotent stromal cells that were first isolated and characterized from bone marrow (BMMSC)^8^. A number of studies have shown MSCs have a great potential to differentiate into epithelial cells^9–11^. As a result, BMMSCs can be used for LSCD treatment as shown in previous animal models^12^. Similarly, limbal niche cells (LNC) are progenitor cells isolated from the corneal limbal niche using collagenase digestion and cultured in modified embryonic stem cell medium (MESCM)^13^ on Matrigel coated plastic surface. LNCs are characterized by a small spindle shape, high growth rate and expression of embryonic stem cell (ESC) markers^12^. LNCs may be induced to differentiate into blood vessel endothelial cells, paracytes, osteoblasts, chondrocytes and adipocytes, expressing MSC markers like CD73, CD90, CD105, thus defined as mesenchymal progenitors^12^. More importantly, LNCs have been shown to more effectively prevent limbal epithelial progenitors from aging compared to BMMSCs^14–17^. However, it is unclear whether LNCs can prevent LSCD, and if so, whether LNCs are better than BMMSCs. In this study we compare the efficiencies between human LNCs and BMMSCs to prevent LSCD, and elucidate their potential mechanism. Herein, our results suggest for the first time that subconjunctivally transplanted LNC are more powerful than BMMSC to prevent LSCD in an alkali burn rabbit model, at least partially, due to activation of SCF-c-Kit signaling.

## Results

### LNCs express higher MSC and neural crest markers than BMMSC

**Figure 1:**
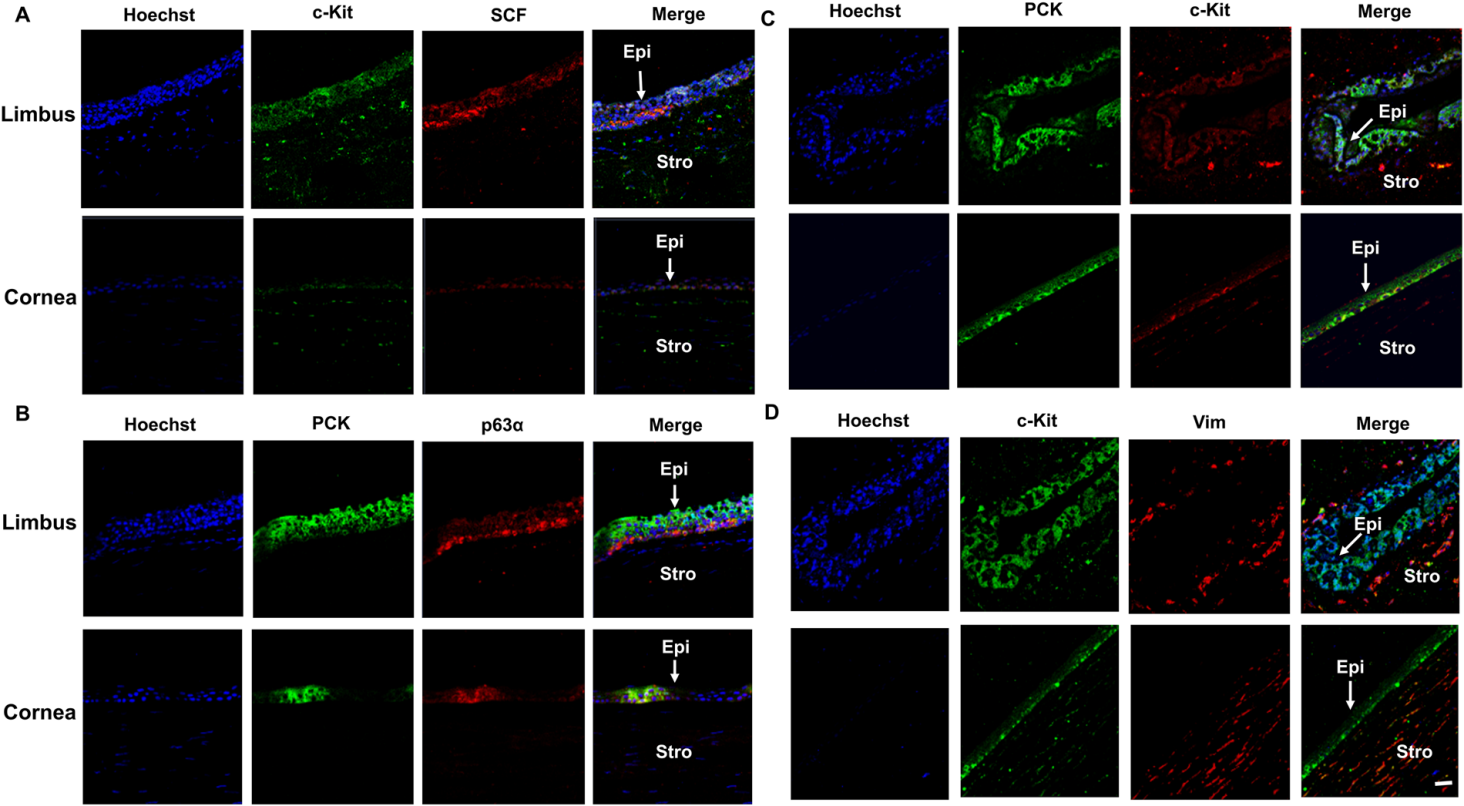
Expression of SCF, C-kit and p63α in Human Limbus and Cornea. Cross sections of normal limbus and cornea were subjected to double immunostaining of C-kit/SCF(**A**), PCK/P63α (**B**), PCK/C-kit (**C**), C-kit/Vim (**D**). Nuclear counterstained by Hoechst 33342. Abbreviations: Epi, epithelium; Stro, stroma. Scale bar = 50 µm.

**Figure 2:**
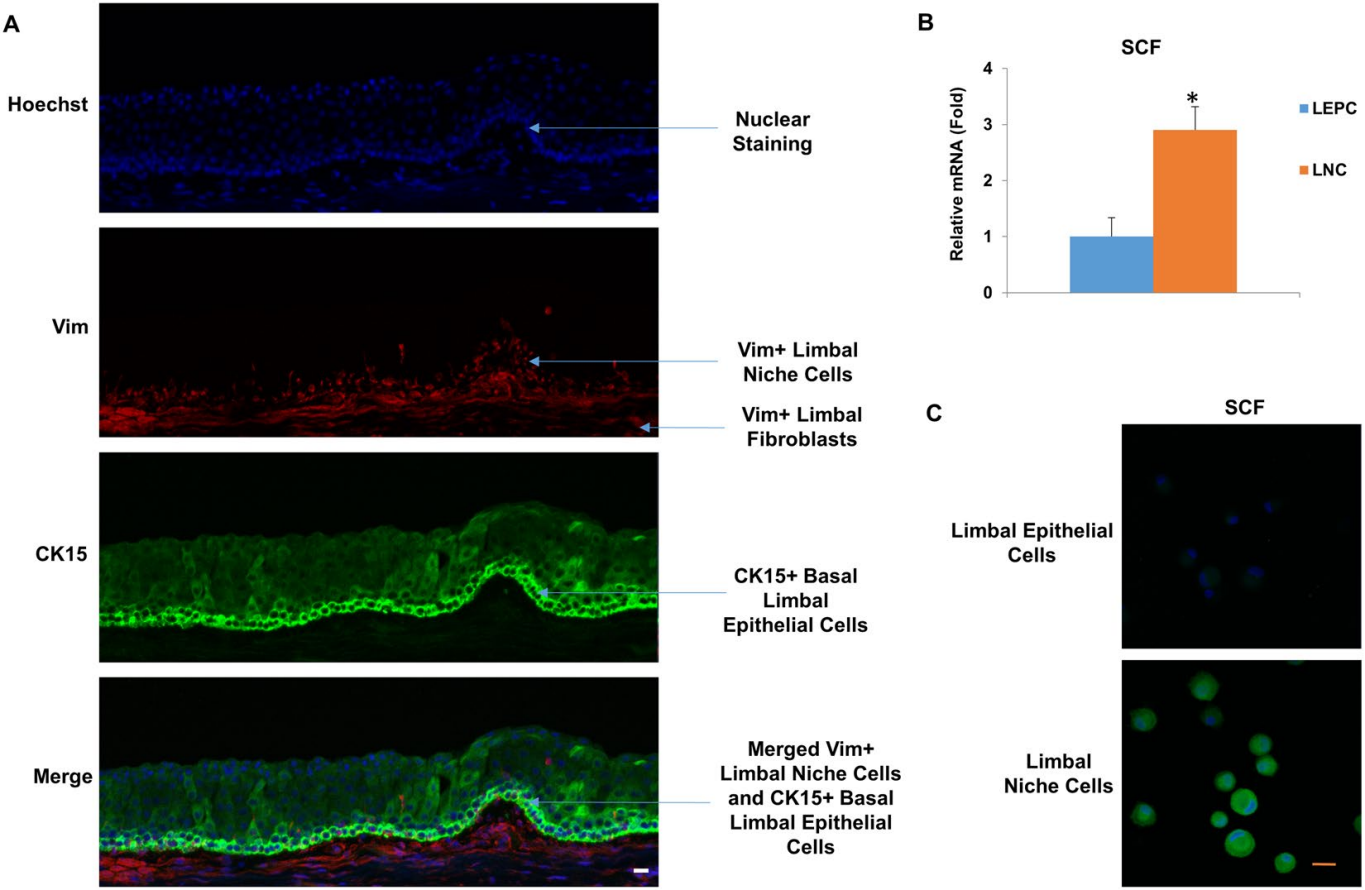
LNCs express more SCF than LEPCs. Cross sections of normal limbus were subjected to immunostaining of Vim and CK15 (**A**). Nuclear counterstained by Hoechst 33342. bar = 50 µm. LEPCs were isolated by dispase overnight digestion at 4 °C and LNCs isolated by collagenase overnight digestion at 4 °C, and then real-time PCR and immunostaining of SCF performed (**B** and **C**). Scale bars = 25 µm.

**Figure 3:**
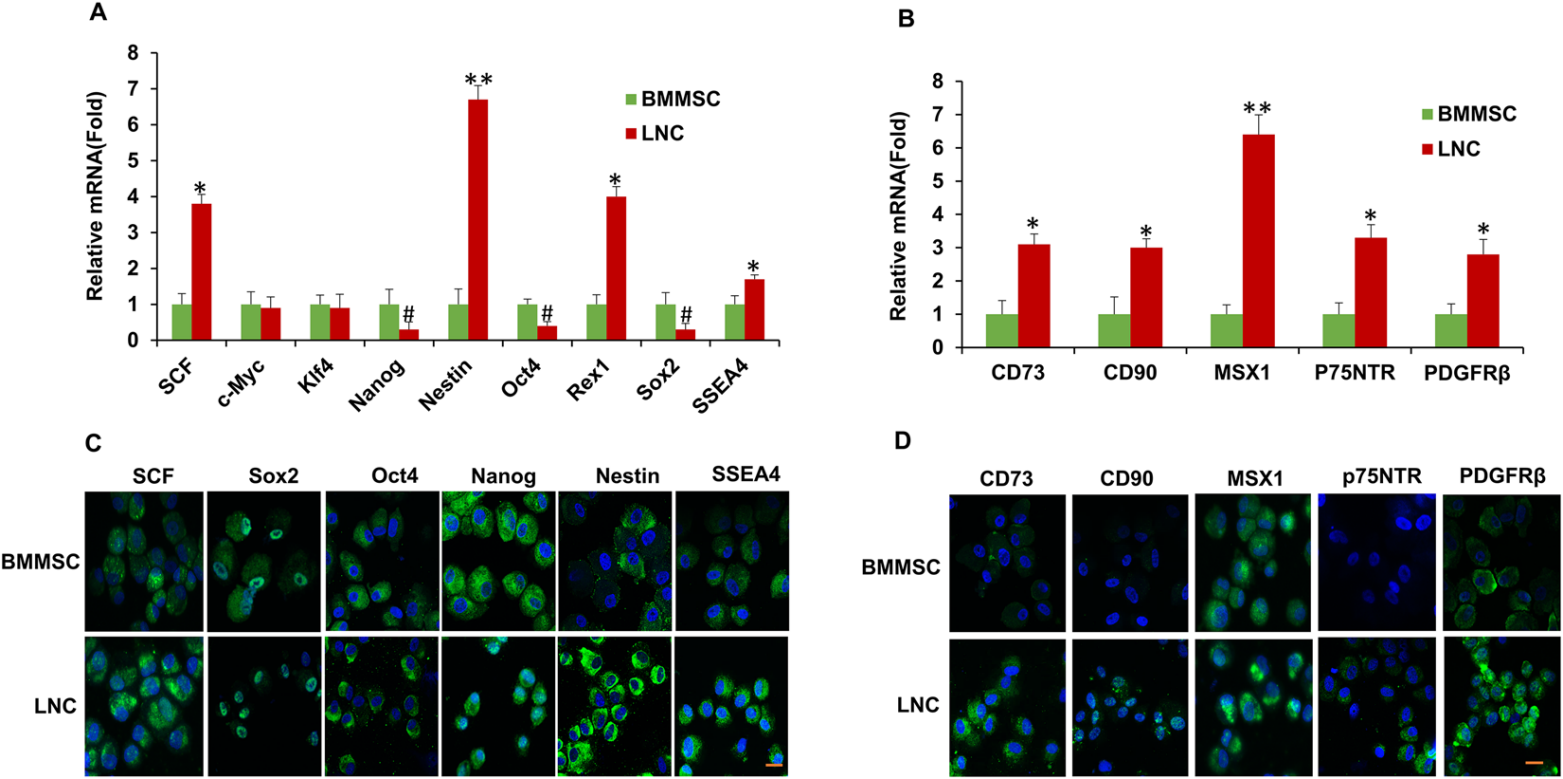
LNC express more ESC, MSC and NC markers. P4 LNC and P4 BMMSC cultured on 2D Matrigel in MESCM were subjected to qRT-PCR for transcription expression of ESC markers (**A**), MSC and neural crest markers (**B**, n = 3, *p < 0.05, ^#^p < 0.05 and **p < 0.01 respectively) and immunostaining of ESC markers (**C**) and MSC, NC markers (**D**). Nuclear counterstained by Hoechst 33342. Scale bars = 25 µm.

**Figure 4:**
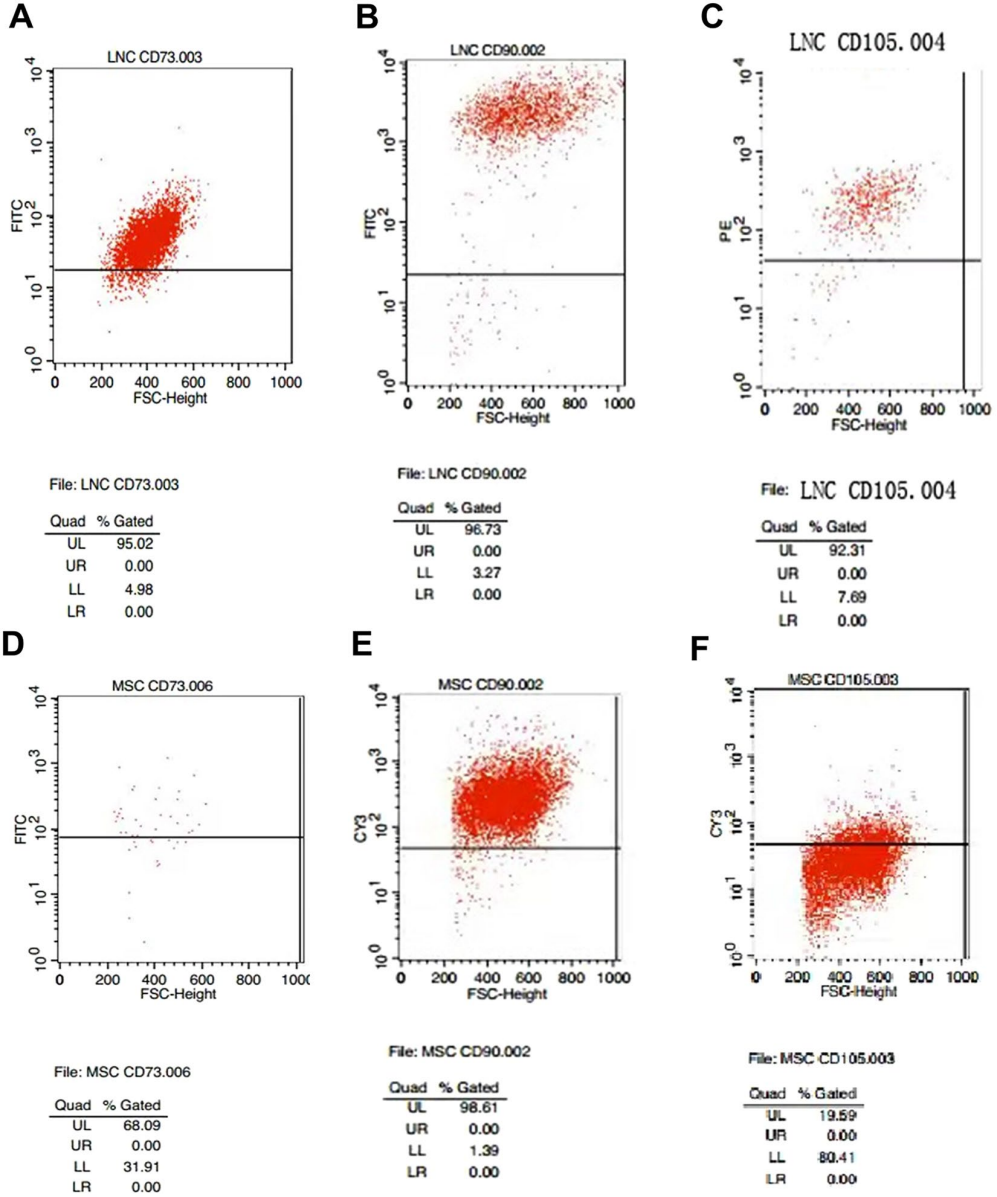
Expression of CD73, CD90, CD105 in LNC and BMMSC. P4 LNC and P4 BMMSC cultured on 2D Matrigel in MESCM were subjected to fluorescence-activated cell sorting (FACS) analysis of MSC markers (**A**–**F**, n = 3).

Anatomically, limbal niche cells (LNC) are located at the palisades of Vogt, of which the epithelium interfaces with basement membrane and consists of intermittent projections^18,19^. As reported^14^, collagenase digestion results in a cluster of cells consisting of both epithelial cells and subjacent mesenchymal cells, of which the later can express ESC markers^17^. In our study, we first removed the epithelial sheet by dispase and then digested the remaining stroma in collagenase. To characterize LNCs and BMMSCs, we double immunostained cornea-limbus sections with pan cytokeratin (PCK), vimentin (Vim) to delineate the epithelium and the stroma in the limbal and cornea region and double immunostained C-kit/SCF (Fig. 1A), PCK/P63α (Fig. 1B), PCK/C-kit (Fig. 1C), and C-kit/Vim (Fig. 1D) to show SCF and c-kit were expressed much higher in the limbus compared to other regions of the cornea in which most SCF was expressed in the basal layer (Fig. 1A). Most PCK+ limbal epithelial cells expressing P63α were also in the basal layer (Fig. 1B). In addition, P63α was positive in the nucleus of basal layers in the limbus but negative in the central cornea (Fig. 1B). C-kit was predominately expressed by epithelial but not stroma layers of both the limbus and cornea (Fig. 1A,C and D). To further resolve the issue of location of LNCs and expression of SCF in LNCs and LEPCs, we have performed immunostaining of Vim (a specific marker of stroma) and CK15 (a specific marker of basal epithelium) to demonstrate that in the basal layer, LNCs are located together with LEPCs (in deep stroma, those Vim+ cells are limbal fibroblasts). In Fig. 2A, we can clearly see limbal epithelial cells and LNCs are merged together in the basal layer. In addition, we have also isolated LEPCs by dispase overnight digestion at 4 °C and LNCs by collagenase overnight digestion at 37 °C, and then performed real-time PCR and immunostaining of SCF, which supports our conclusion that LNCs express much more (3X) SCF than LEPCs (Fig. 2B and C). Further analysis showed BMMSCs expressed more ESC markers such as Nanog (3X), Oct4 (2X) and Sox2 (2X), however expressed significantly less ESC markers such as Nestin (7X), Rex1 (4X) and SSEA4 (2X), MSC markers such as CD73 (3X), CD90 (3X), CD105 (2X), and NC markers such as MSX1 (6X), P75NTR (3X) and PDGFRβ (3X) (Fig. 3A and B). These data suggest LNC are indeed a powerful source of progenitors in the local limbal tissue. The immunostaining results showed a similar trend of their corresponding protein expression, supporting the results from real-time PCR (Fig. 3C and D). To further confirm our findings, we used flow cytometry to analyze surface antigen characteristics. The results indicated surface antigens that are characteristic to MSCs, including CD73, CD90, CD105, were expressed in both LNCs and MSCs. The percentage of LNCs which express CD73, CD90, CD105 and SCF was approximately 95%, 97%, 92% and 11%, respectively, in contrast to that of BMMSC which was 68%, 99%, 20% and 3%. This shows LNCs express significantly higher levels of MSC positive markers CD73, CD105 and cytokine SCF (p < 0.01) and a similar level of CD90 (p > 0.05) compared to those in BMMSC (Fig. 4).

### LNCs express higher SCF than BMMSC

**Figure 5:**
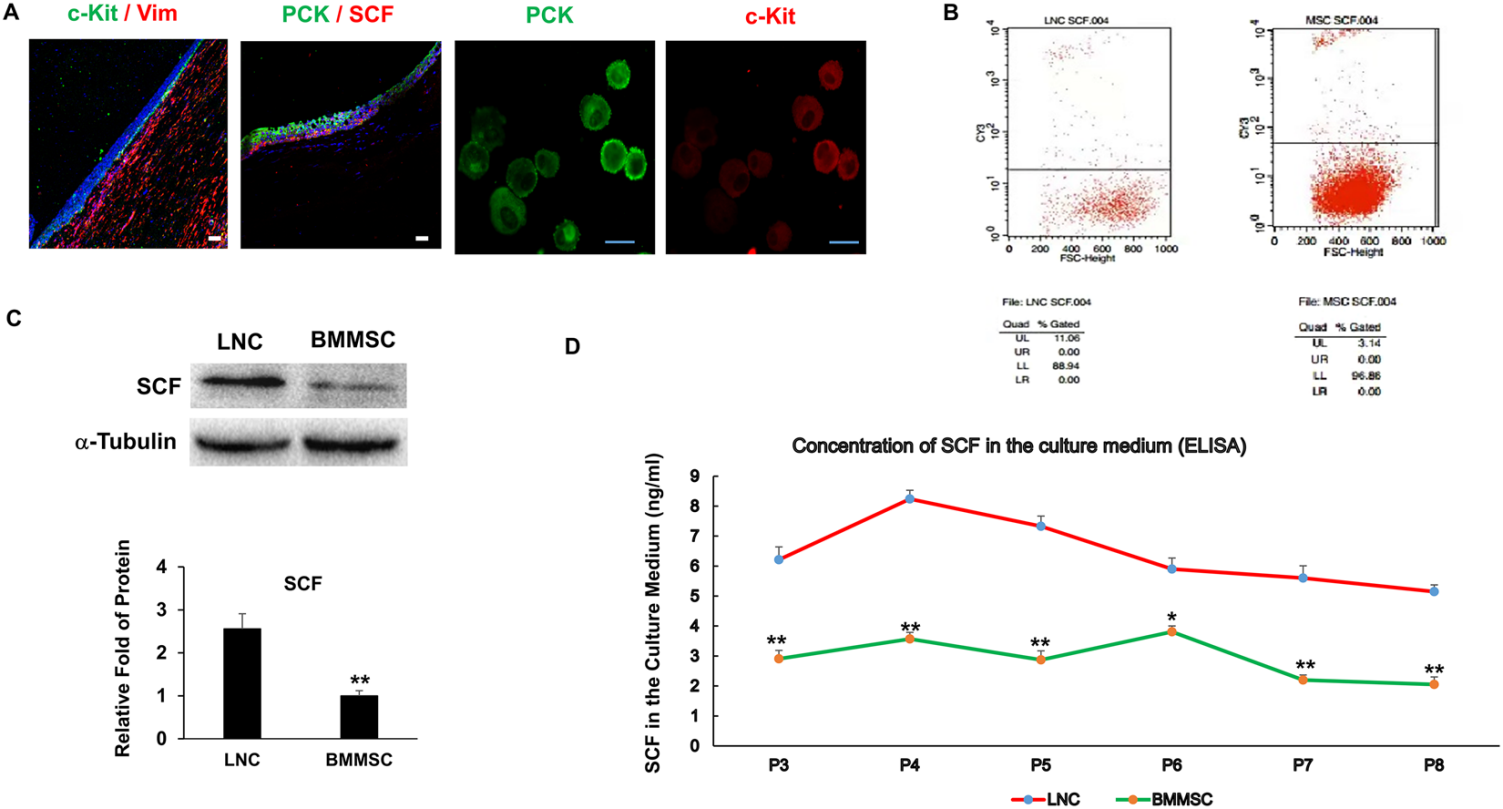
LNC expresses higher SCF than BMMSC. Cross sections of normal limbus and fresh isolated epithelial cells were subjected to double immunostaining of C-kit/Vim, PCK/SCF and PCK/C-kit (**A**, nuclear counterstained by Hoechst 33342, scale bar = 25 µm), P4 LNC and P4 BMMSC cultured on 2D Matrigel in MESCM were subjected to FACS analysis of SCF (**B**), western blot analysis of SCF (**C**, using α-Tubulin as the loading control, n = 3, **p < 0.01), the culture media of LNC and BMMSC from P3 to P8 were collected and subjected to ELISA analysis (**D**).

As noted previously, the haematopoietic stem cell marker c-Kit, also known as CD117, is important to support pancreatic beta cell proliferation, maturation and survival^20–22^. Through binding to its legend SCF (also known as steel factor), c-Kit can induce subsequent cell proliferation, differentiation, survival and migration^23,24^. To explore whether SCF-c-Kit signaling was involved in our experiment and to compare the differences between LNCs and BMMSCs, we characterized SCF and c-Kit by *in vivo* cytolocalization. Double immunostaining of human limbal sections showed positive c-Kit staining in Vim- but not Vim+ stromal cells (Fig. 5A). In contrast, strong SCF staining was found in PCK- stromal cells located very close to the basal layer of epithelial cells (Fig. 5A). To confirm whether a difference indeed existed in limbal epithelial cells and niche cells (NC), limbal epithelial sheets were separated using Dispase and released by trypsin/EDTA. Double immunostaining confirmed that c-Kit was expressed only by PCK+ epithelial cells (Fig. 5A). Flow cytometry showed that 11.06% of the LNC were SCF-positive in contrast to 3.14% of the BMMSC (p < 0.01, Fig. 5B), suggesting that the extent of activation of SCF-c-Kit signaling in LNCs was much greater than that in BMMSCs. In addition, ELISA showed LNCs excreted more SCF than BMMSC from passage 3 to passage 8 (p < 0.01, Fig. 5D), confirming activation of SCF-c-Kit signaling in LNCs was more significant than BMMSCs. In addition, RT-real time PCR confirmed LNCs expressed 4 times more SCF than BMMSCs (Fig. 3A). Furthermore, Western blot result also showed the expression of SCF was 2.5-fold higher than that in BMMSC (p < 0.01, Fig. 5C), suggesting that LNCs express higher SCF than BMMSC.

### Knockdown of SCF in LNCs induces loss of niche function

**Figure 6:**
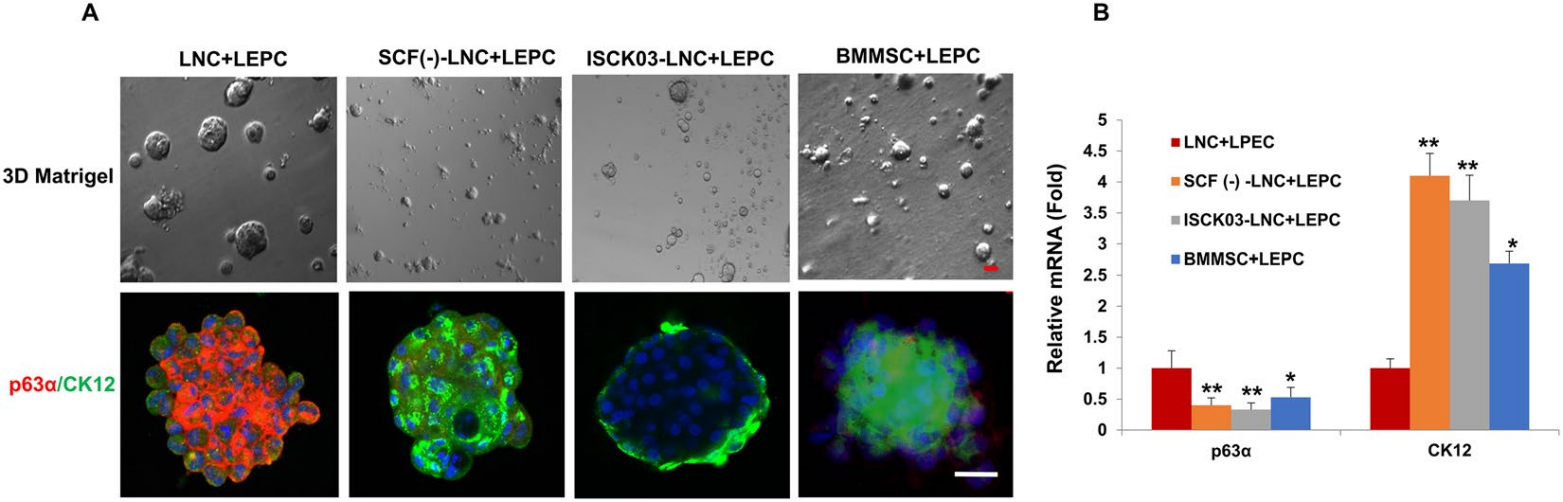
Knockdown of SCF and blockage of c-kit in LNC induced loss of niche function for LSC. Single limbal epithelial progenitor cells (LEPC) were co-cultured with LNC, SCF(−)-LNC (SCF knock-down LNC), c-Kit inhibitor ISCK03 (Santa Cruz Biotechnology) or BMMSC on 3D Matrigel in MESCM for ten days before subjected to morphological analysis by phase-contrast microscopy (**A**, upper panel, scale bar = 25 µm) and immunostaining of P63α and CK12 (**A**, lower panel, nuclear counterstained by Hoechst 33342, scale bar = 25 µm) and qRT-PCR for transcription expression of P63α and CK12 (**B**, n = 3, *P < 0.05 and **P < 0.01,respectively).

Previously it was reported that LNCs and LSCs could yield sphere growth on 3D Matrigel in MESCM, which prevented LSCs from differentiation^25^. To determine whether LNC could yield more sphere growth and maintain the stemness of LSCs better than BMMSC, we co-cultured LEPC with either LNCs, SCF(−)-LNCs (SCF knock-down LNC), LNCs with c-Kit inhibitor ISCK03, or BMMSCs for ten days. We then viewed their morphology under phase contrast microscopy and used double immunostaining with P63α/CK12. Bigger spheres were shown in the LNC group while the smallest spheres were found in the SCF(−)-LNC and c-Kit ISCK03 groups (Fig. 6A, upper line). Accordingly, the relative mRNA level of P63α was highest but CK12 was the least in the LNC group while the SCF(−)-LNC and ISCK03 groups had the least P63α but the highest CK12 (Fig. 6A, bottom line and Fig. 6B). This suggests knockdown of SCF or inhibition of SCF-c-Kit signaling in LNCs induces loss of their niche function.

### LNC transplantation prevents LSCD, better than BMMSC

**Figure 7:**
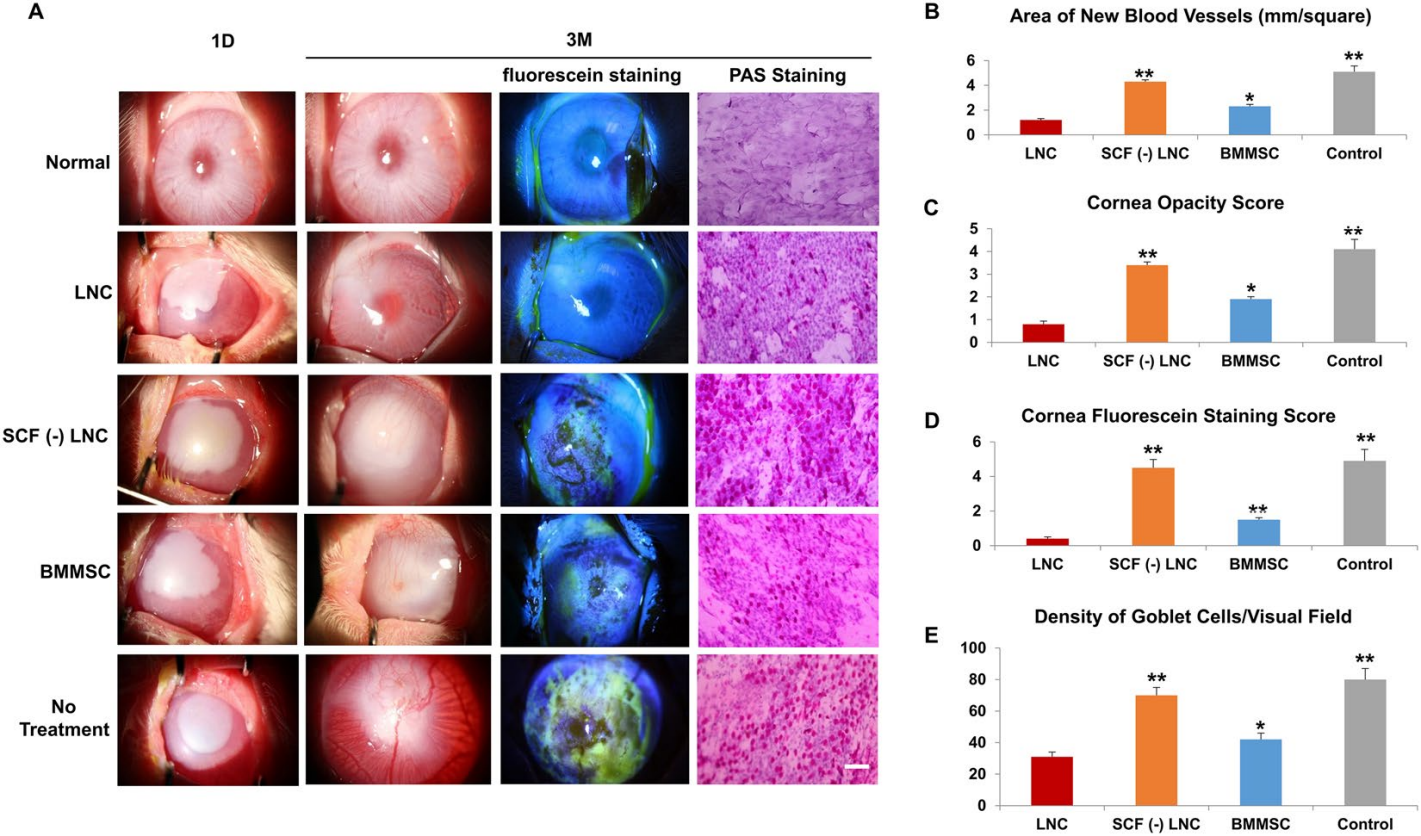
LNC transplantation prevent limbal stem cell dysfunction better than BMMSC. Normal rabbit corneas were burn with 1 M potassium hydroxide solution for thirty seconds before subjected to photography analysis (**A**, first column, using normal rabbit cornea as the control). After 3 months with different treatment (cell transplantation), the rabbit corneas were subjected to photography analysis again (**A**, second column), with fluorescein staining (**A**, third column). The filtration membrane on rabbit corneas were subjected to PAS staining (**A**, last column). The area of blood vessels (**B**), cornea opacity score (**C**), cornea fluorescein staining score **(D**) and density of PAS + conjunctival goblet cells (**E**) were analyzed. *p < 0.05 and **p < 0.01 respectively. N = 3, Scale bar = 25 µm.

**Figure 8:**
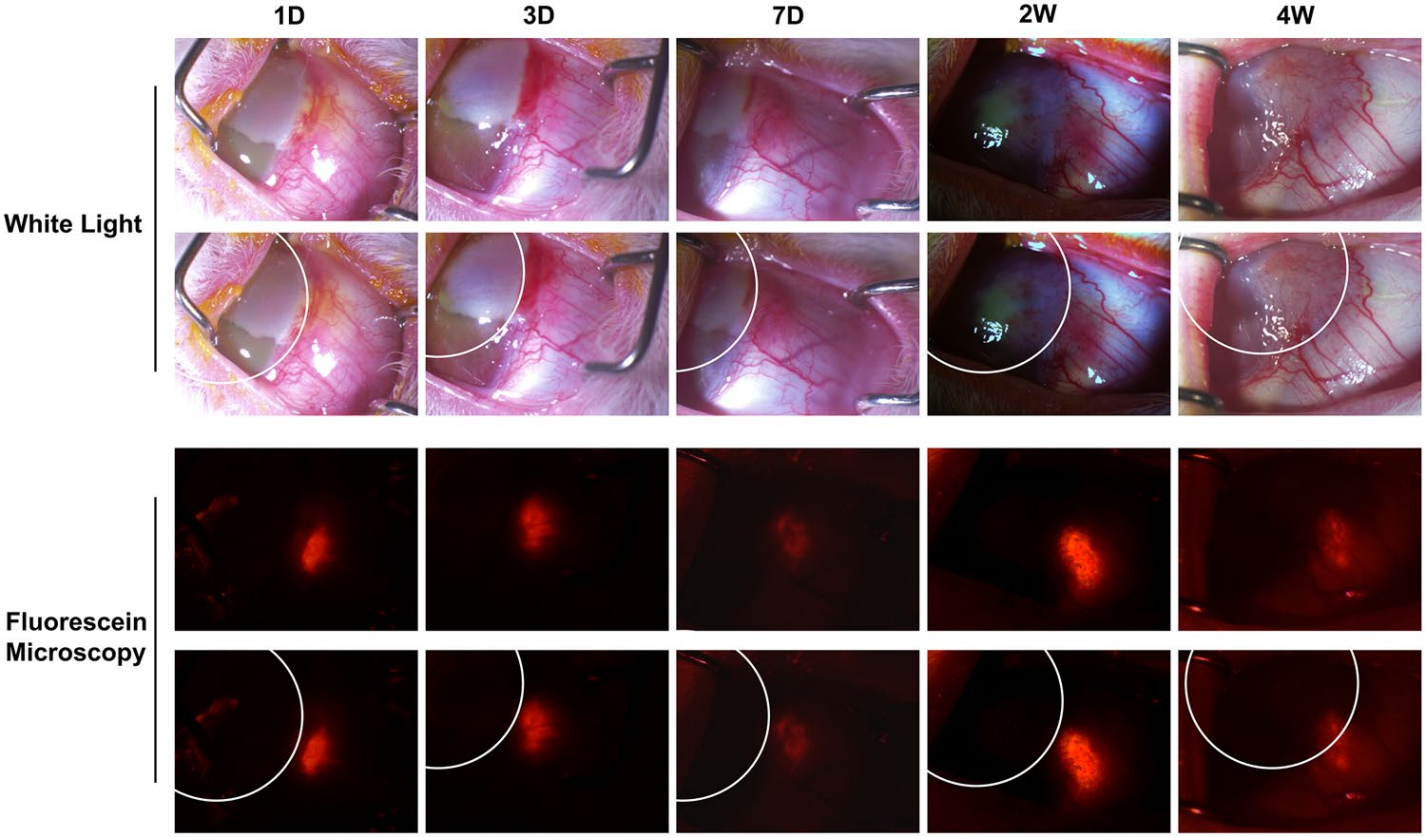
Distribution of LNC after subconjunctival transplantation. The LNC cells were pre-labeled with CellTracker™ CM-DiI before transplantation (red fluorescein when viewed under fluorescein microscopy) and tracked *in vivo* 1 day, 3 days, 7days, 2 weeks, 4 weeks after transplantation. The while cycle line was used for analysis to indicate the edge of limbus. N = 3.

Clinical characterization for LSCD includes corneal opacity, persist epithelial defects, neovascularization and high goblet cell density in cornea^11,12,26^. In our rabbit model, alkali burn without treatment (control group) developed severe cornea opacity, neovascularization, and conjunctivalization of the corneal surface within two weeks. Three months after transplantation, the rabbits in the control group retained significant LSCD (Fig. 7). In contrast, the rabbits from both the LNC and BMMSC treated groups recovered significantly from LSCD (Fig. 7). In addition, the eyes of rabbits in the LNC group were better recovered than those in the BMMSC group (Fig. 7). Other clinical features are presented in Table 1. Compared to the BMMSC group, the LNC group had a lower corneal opacity score (p < 0.05), less neovascularization (p < 0.05), a lower corneal fluorescein staining score (p < 0.01), and a lower goblet cell density in cornea (p < 0.05). PAS staining demonstrated that goblet cells were absent in normal rabbit cornea but appeared after alkali burn (Fig. 7) and LNC treated group had significantly fewer goblet cells than BMMSC group, suggesting that LNCs protect LSCs better than BMMSCs. Interestingly, the results from the SCF (−) LNC group were similar to those of the control group. Furthermore, we also observed the movement of cells from the limbus to the cornea using CM-DiI labeled LNC. Four weeks after transplantation, the movement of cells was clearly visualized towards the cornea under fluorescein microscopy (Fig. 8), suggesting that LNC transplantation prevented LSCD, better than BMMSCs.

**Table 1:** Comparison of LSCD three months after LNC and BMMSC treatment.

Treatment	n	Corneal-opacity score	Neovascularization scales (mm^2^)	Corneal fluorescein staining score	Goblet cell density in cornea (number/mm^2^)
Control	3	2.67 ± 0.58	25 ± 5	2.33 ± 0.58	72 ± 19.7
LNC	4	0.50 ± 0.58	5.5 ± 3	0.25 ± 0.5	14.25 ± 7.1
BMMSC	5	1.60 ± 0.55	11.2 ± 3.11	1.2 ± 0.45	32.8 ± 9.2
P value		0.022^*^	0.027^*^	0.002^**^	0.013^*^

*p < 0.05 and **p < 0.01 respectively.

## Discussion

MSCs refer to a group of multipotent stromal cells that were first isolated and characterized from the bone marrow^8^ but have now been found from nearly all adult tissues^27^. A number of studies have disclosed that MSC have a great potential in regenerative medicine due to their unique properties of self-renewal, high plasticity, modulation of immune responses, and flexibility for genetic modification^9–12,28^. Bone marrow derived MSCs (BMMSC) have been well characterized and widely used in multiple clinical trials^10,29^. They have also been used to prevent LSCD after alkali burn in rats and rabbit models, through the former function or differentiation into cornea epithelial cells^29,30^. As previously reported, we have successfully isolated human limbal niche cells (LNC) by digesting the limbal stroma with collagenase after removing the epithelium with dispase^14,15^ and proved that the stemness of limbal stem cells/epithelial progenitor cells (LSC/LEPC) could be maintained during culture with LNC in a 3D environment^17^. Herein, we report that LNCs express more ESC markers (such as Nestin, Rex1 and SSEA4), MSC markers (such as CD73, CD90, CD105), NC markers (such as MSX1, P75NTR and PDGFRβ and cytokine SCF), but less Nanog, Oct4 and Sox2 than BMMSCs (Figs 1–5). These results suggest LNCs are likely a better source of progenitors in the limbal tissue than BMMSCs. In addition, LNCs and BMMSCs differ in some other key markers and biological properties. For example, we have demonstrated that SCF and c-Kit were expressed in the limbal region. SCF, a product of the Steel locus in mouse^31^, is widely expressed in the body by endothelial cells, fibroblasts, and stromal cells^32^. C-Kit, also known as SCF receptor or CD117, is a cellular homologue of the v-Kit oncogene. C-Kit protein has been found in a wide range of cells and tissues including mast cells^33,34^, melanocytes^35^, vascular endothelial cells^36^, interstitial cells of Cajal^37^, testis^34^ and of course, bone marrow^38^. The binding of SCF to c-Kit plays an important role in migration, proliferation and survival in multiple cell types^17,39^. In our study, expression of SCF mRNA and protein is significantly higher in LNCs than that in BMMSCs *in vivo* (Fig. 2) and *in vitro* (Fig. 3), suggesting LNCs are probably a better source of progenitors in the limbus. Consequently, significantly higher protein expression and secretion of SCF are observed in LNCs than in BMMSCs (Fig. 5), supporting that LNCs have more active SCF-c-Kit signaling than BMMSCs (Fig. 5). Interestingly, when cultured with LSCs on 3D Matrigel in MESCM, LSC could be reunited not only with LNCs but also BMMSCs (Fig. 6). However, such reunited LSCs expressed significantly higher SC markers such as p63α but significantly lower differentiation markers such as CK12 (Fig. 6), demonstrating that LNCs are more supportive than BMMSC. To demonstrate that LNCs are more supportive to LSCs than BMMSCs, we used SCF siRNA to attenuate expression of SCF in LNCs to block SCF-c-Kit signaling. As expected, attenuation of SCF-c-Kit signaling by SCF siRNA or c-Kit inhibitor ISCK03 was associated with the loss of LSC stemness in LNCs (Fig. 6). Furthermore, LSC reunited with BMMSC showed a less active SCF-c-Kit signaling than with LNC (Fig. 6), indicating that LNC could promote the stemness of LSCs better than BMMSCs, which is mediated at least partially through SCF-c-Kit signaling.

Because LNCs seems be more progenitor than BMMSC, we wondered whether LNCs might be more effective for specific therapeutic applications, for example, treatment of limbal stem cell deficiency (LSCD) and reconstruction of corneal surface. The hallmark of LSCD is persistent corneal epithelial defect and conjunctivalisation of the cornea. Depending on the extent of limbus involved, LSCD can be divided into partial or total deficiency. The main features of LSCD include (a) limbal structural disorder, (b) epithelial defects even ulceration, (c) abnormal epithelium, (d) neovascularization, (e) scarring and keratinization, and (f) unstable tear film^40–45^. In this report, we demonstrate that rabbit eyes in the LNC treatment group had a lower corneal opacity score, neovascularization, corneal fluorescein staining score, and goblet cell density compared to that in the BMMSC group 3 months after alkali burn (Fig. 7). This demonstrates LNCs are more supportive for LSCs than BMMSCs, probably due to activation of SCF-c-Kit signaling (Figs 6 and 7). Interestingly, we observed the movement of LNCs to the cornea (Fig. 8). Our findings represent an encouraging method for treating cornea alkali burn and other ocular surface diseases caused LSCD by transplantation of LNC (and also transplantation of BMMSCs if LNCs are not available). Further studies are required for clarification of the exact mechanistic interaction of LNCs with native corneal cells from the limbus. Our findings suggest for the first time that subconjunctivally transplanted LNCs are more powerful than BMMSCs in an alkali burn rabbit model to prevent limbal stem cell deficiency, at least partially, due to SCF-c-Kit signal activation.

## Methods

### Materials

Corneoscleral rims from 18 to 60 years old donors were obtained from Wuhan Red Cross Eye Bank in Tongji hospital (Wuhan, China) and managed in accordance with the declaration of Helsinki. The identities of these anonymous cadaver donors could not be identified. The research protocol and the rabbit model protocol were approved by the Institutional Research, Animal Care and Use Committee of the University of Huazhong University of Science and Technology, Wuhan, China.

### Cell isolation and culture

Human LNC were isolated and cultured as previously prescribed^14–17^. Each corneoscleral rim was cut into 4 average pieces and digested with Dispase II at 4 °C for 16 h to generate intact epithelial sheets^15,18^, after remove of the epithelial sheets, the remaining stroma were further digested in collagenase A (Coll) at 37 °C for 18 h to generate limbal niche clusters^13–15,17^. The clusters were further digested with 0.25% trypsin and 1 mM EDTA (T/E) at 37° for 5 min to yield single cells before being seeded at the density of 1 × 10^4^ per cm^2^ on Matrigel coated 6-well plates in MESCM (ESCM containing 10 ng/ml LIF and 4 ng/ml bFGF)^13^. Upon 80–90% confluence, cells were passaged at the density of 5 × 10^3^ per cm^2^. The second passage of bone marrow-derived MSC (BMMSC, PT-2501) was obtained from LONZA (Allendale, NJ) and cultured in parallel.

### Culture on 3D Matrigel

Three dimensional (3D) Matrigel was prepared by adding 150 μl of 50% Matrigel (diluted in MESCM) per chamber of an 8-well chamber slide following incubation at 37 °C for 1 h. Cells were seeded on 3D Matrigel and cultured for 10 days in MESCM. As reported^15,17,19^, single limbal epithelial progenitor cells obtained by Dispase-isolated limbal epithelial sheets were mixed at a ratio of 4:1 with the 4^th^ to 6^th^ passage LNC cells or BMMSC at the total density of 5 × 10^4^ per cm^2^. After 10 days of culture in MESCM, the resultant sphere growth was collected by digestion of Matrigel with 10 mg/ml dispase II at 37 °C for 2 h.

### siRNA transfection

For the siRNA knockdown, the 4^th^ to 6^th^ passage LNC cells were subjected to 48 hours of transfection by mixing 50 µl of serum-free medium with 1 µl of HiPerFect siRNA transfection reagent (final dilution, 1:300) and 1 µl of 20 µM scRNA (as the control) or siRNA to SCF (ThermoFisher, Waltham, MA) each at the final concentration of 100 nM, drop-wise, followed by culturing the cells in fresh medium at 37 °C^20^.

### RNA extraction, reverse transcription and real-time PCR

Total RNAs were extracted using RNeasy Mini Kit and reverse-transcribed using High Capacity Reverse Transcription Kit. cDNAs were amplified by real-time RT-PCR using specific primer-probe mixtures and DNA polymerase in 7300 Real Time PCR System (Life Technologies). Real-time RT-PCR profile consisted of 10 min of initial activation at 95 °C, followed by 40 cycles of 15 sec denaturation at 95 °C, and 1 min annealing and extension at 60 °C. The relative gene expression data were analyzed using the comparative CT method (ΔΔCT). All assays were performed in triplicate. The results were normalized by glyceraldehyde 3-phosphate dehydrogenase (GAPDH) as an internal control.

### Impression cytometry and Periodic Acid Schiff (PAS) staining for goblet cells

After impression of the filtration membrane on rabbit corneas, the filtration membrane was deparaffinized and hydrated to water, oxidized in 0.5% periodic acid solution, rinsed and placed in Schiff reagent, washed again, and counterstained in Mayer’s hematoxylin. The filtration membrane was dehydrated and mounted with coverslips using a synthetic mounting medium, and examined under a microscope. The goblet cells were stained red.

### Immunofluorescence staining

Normal human limbus was fixed using paraformaldehyde, prepared for cross section and immunofluorescence stained as described below. Single cells were prepared for cytospin using Cytofuge® at 1,000 rpm for 8 min (StatSpin, Inc., Norwood, MA), fixed with 4% paraformaldehyde for 15 min, permeabilized with 0.2% Triton X-100 in PBS for 15 min, and blocked with 2% BSA in PBS for 1 h before being incubated with primary antibodies overnight at 4 °C. After washing with PBS, cytospin preparations were incubated with corresponding secondary antibodies for 1 h using appropriate isotype-matched non-specific IgG antibodies as controls. The nucleus was counterstained with Hoechst 33342 before being analyzed with a Zeiss LSM 700 confocal microscope (LSM700, Carl Zeiss. Thornhood, NY).

### Examination of SCF and MSC markers with flow cytometry

The expression of SCF, CD73, CD90 and CD105 in LNCs and BMMSCs were analyzed with flow-cytometry. Cells were collected and stained with already-labeled antibodies (SCF, CD70, CD90 and CD105, 1:50 dilution) for 15 minutes at room temperature in the blocking buffer (3% BSA and 0.05% Tween-20 in PBS). Fluorescence-activated cell sorting (FACS) analysis was performed using Becton Dickinson LSRII, FACS Diva software (BD, San Jose, CA) and FlowJo software (Tree Star, Ashland, OR). For each sample, 10,000 events were recorded, and live cells were gated and analyzed.

SCF specific antibody has been pre-coated onto 96-well plates and blocked (ab108901, Abcam, Cambridge, MA). Standards or test samples are added to the wells and subsequently a SCF specific biotinylated detection antibody is added and then followed by washing. Streptavidin-Peroxidase Conjugate is added and TMB is then used to visualize Streptavidin-Peroxidase enzymatic reaction. The density of yellow coloration is directly proportional to the amount of SCF captured in plate following the manufacturer’s instructions.

### ELISA

SCF ELISA kit was obtained from ThermoFisher Scientific (EHKITLG, Waltham, MA). The experiments were performed following the instructions of the manufacturer.

### Western blotting

Proteins were extracted in RIPA buffer supplemented with proteinase inhibitors. Equal amounts of proteins determined by the BCA assay (Pierce, Rockford, IL) in total cell extracts were separated by 10% SDS-PAGE and transferred to nitrocellulose membranes. Membranes were then blocked with 5% (w/v) fat-free milk in TBST (50 mM Tris-HCl, pH 7.5, 150 mM NaCl, 0.05% (v/v) Tween-20), followed by sequential incubation with specific primary SCF antibody and their respective secondary antibody using α-tubulin as the loading control. The immunoreactive bands were visualized by a chemiluminescence reagent (Pierce, Dallas, TX).

### Rabbit alkali-burn LSCD model

Under topical anesthesia using 0.5% pimecaine hydrochloride eye drops for 3 times, round filter paper of 4 mm in diameter soaked with 1 M potassium hydroxide solution were placed to the upper temporal peripheral corneal for 30 seconds and rinsed with saline, 100 ml each eye. The degree of corneal opacity, epithelial defect area, neovascularization area, corneal fluorescein staining score and corneal goblet cell density were recorded and quantified according to the published method to access the severity of LSCD at the first day, 7 days, 2 weeks, 4 weeks, 3 months of observation^21^.

### Transplantation and ***in vivo*** tracking of LNC and BMMSC

Transplantation method involved 5 × 10^3^ cells/0.2 ml, marked with CellTracker™ CM-DiI, transplanted by subconjunctival injection, immediately after alkali burn.

### Statistical analysis

All data were repeated at least 3 times independently, and reported as means ± SD, calculated for each group and compared using ANOVA and the Student’s paired t-test by Microsoft Excel (Microsoft, Redmont, WA). Test results were reported as two-tailed p values, where p < 0.05 was considered statistically significant.

### Data availability

All the data are available for tracking.
